# Downregulation of Roundabout guidance receptor 2 suppresses hepatocellular carcinoma progression by interacting with Y-box binding protein 1

**DOI:** 10.1038/s41598-024-53013-3

**Published:** 2024-01-31

**Authors:** Ting Liu, Congjie Zhai, Bo Tian, Chao Li, Shuangshuang Han, Shihui Wang, Mingda Xuan, Dehua Liu, Yunxia Zhao, Hongyan Zhao, Weifang Yu, Jia Wang

**Affiliations:** 1 Department of Endoscopy Center, The First Hospital of Hebei Medical University, No. 89 Donggang Road, Shijiazhuang, 050031 Hebei China; 2 Hebei Key Laboratory of Colorectal Cancer Precision Diagnosis and Treatment, The First Hospital of Hebei Medical University, No. 89 Donggang Road, Shijiazhuang, 050031 Hebei China; 3 Department of Infectious Diseases, The First Hospital of Hebei Medical University, No. 89 Donggang Road, Shijiazhuang, 050031 Hebei China

**Keywords:** Cancer, Oncogenes

## Abstract

Roundabout guidance receptor 2 (Robo2) is closely related to malignant tumors such as pancreatic cancer and liver fibrosis, but there is no relevant research on the role of Robo2 in HCC. The study will further explore the function and mechanism of Robo2 and its downstream target genes in HCC. Firstly, Robo2 protein levels in human HCC tissues and paired adjacent normal liver tissues were detected. Then we established HepG2 and Huh7 hepatoma cell lines with knock-down Robo2 by transfection with lentiviral vectors, and examined the occurrence of EMT, proliferation and apoptosis abilities in HCC cells by western blot, flow cytometry, wound healing assay and TUNEL staining. Then we verified the interaction between Robo2 and its target gene by Co-IP and immunofluorescence co-staining, and further explored the mechanism of Robo2 and YB-1 by rescue study. The protein expression level of Robo2 in HCC was considerably higher than that in the normal liver tissues. After successfully constructing hepatoma cells with knock-down Robo2, it was confirmed that down-regulated Robo2 suppressed EMT and proliferation of hepatoma cells, and accelerated the cell apoptosis. High-throughput sequencing and validation experiments verified that YB-1 was the downstream target gene of Robo2, and over-expression of YB-1 could reverse the apoptosis induced by Robo2 down-regulation and its inhibitory effect on EMT and proliferation. Robo2 deficiency inhibits EMT and proliferation of hepatoma cells and augments the cell apoptosis by regulating YB-1, thus inhibits the occurrence of HCC and provides a new strategy for the treatment of HCC.

## Introduction

Hepatocellular carcinoma (HCC) is an universal malignant tumor, with high morbidity and mortality, which seriously threatens human health^1^. Although tremendous progress has been attained in the prevention, diagnosis and treatment of tumors in the past fifty years, at present there is still no sufficient and effective treatment due to late diagnosis and the lack of clinical strategies for inhibition of metastasis and promotion of apoptosis^2–5^. Most HCC patients are in advanced stage at the time of diagnosis, which is not suitable for ablation, surgical resection or liver transplantation. In this case, only systemic treatment can be performed^6^. Hence, it is urgent to explore the specific etiopathology of HCC, so as to provide a strong theoretical basis for the early detection, diagnosis and treatment of HCC.

Roundabout guidance receptor 2 (Robo2) protein is a single transmembrane receptor belonging to immunoglobulin superfamily^7,8^. Robo2 protein interacts with numerous intracellular proteins or protein kinases through its four short, conserved sequence motifs: CC0, CC1, CC2, CC3^7,8^. Studies have confirmed that Robo2 protein plays a vital role in pancreatic cancer, glioblastoma and other malignant tumors, such as the loss of Robo2 expression in mouse models of pancreatitis and pancreatic ductal adenocarcinoma (PDAC), and Robo2 plays the role of matrix suppressor gene by inhibiting myofibroblast activation and T cell infiltration^9^; Robo2 inhibits the malignant biological behavior of PDAC, and maybe serve as a predictive biomarker and molecular target of PDAC^10^; Slit2 promotes chemotaxis and polarization of tumor-associated microglia/macrophages through the activation of phosphatidylinositol-3 kinase γ (PI3K-γ) mediated by Robo2^11^.

However, there are still few studies on the performance of Robo2 in liver fibrosis, for example, Zeng et al. clarify that Slit2/Robo2 mediates the occurrence of liver fibrosis and regulates the biological behavior of hepatic stellate cells (HSCs) by motivating PI3K/Akt signaling pathway^12^; soluble triggering receptor expressed on myeloid cells-1 (STREM-1)/Robo2 can promote the activation of HSCs and liver fibrosis through PI3K/Akt and Smad2/3 signaling pathways^13^. At present, there are still no relevant literature report on the function and mechanism of Robo2 in HCC. Therefore, we will deeply probe into the pathogenic mechanism of Robo2 in HCC, providing theoretical basis for elucidating the pathogenesis of HCC, and exploring novel targets for the treatment of HCC.

## Results

### Robo2 is increased in HCC tissues

**Figure 1:**
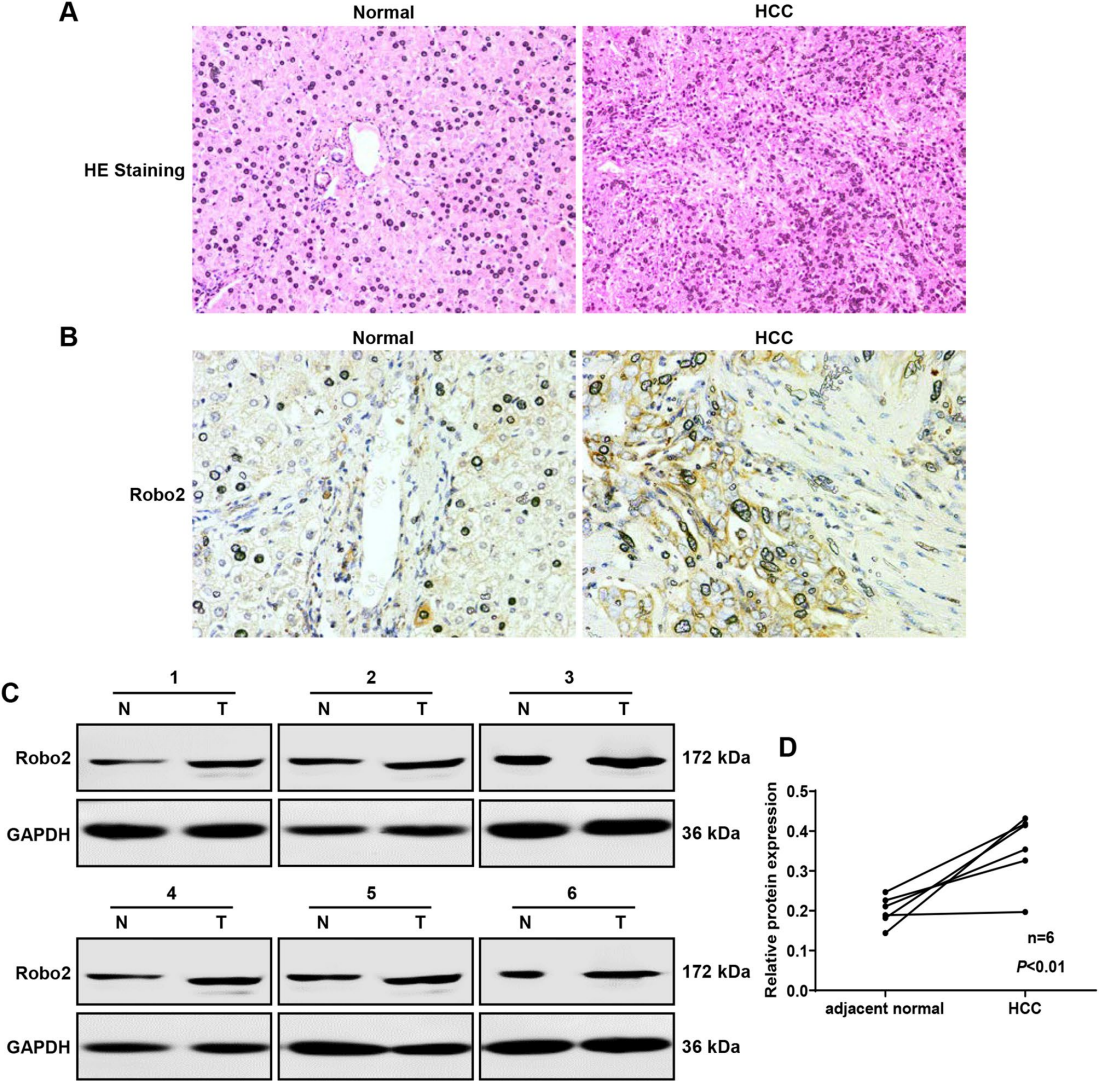
Elevated expression of Robo2 in the HCC tissues. Paraffin-embedded sections were stained with HE (**A**) and IHC staining of Robo2 (**B**). (**C**, **D**) The protein expression levels of Robo2 in cancer tissues and adjacent normal liver tissues of HCC patients were examined by western blot assay. Robo2 protein levels (normalized to GAPDH) were measured by scanning densitometry. The samples derive from the same experiment and that blots were processed in parallel. Original blots are presented in Supplementary Fig. 2.

To clarify the cellular localization and expression of Robo2, IHC staining of Robo2 was performed in human HCC and corresponding normal liver tissues. The consequences showed that Robo2 was mainly distributed on cell membrane and nucleus, and its expression in HCC was strongly positive, considerably higher than that in adjacent tissues (Fig. 1A, B). To further quantitatively analyze the protein expression level of Robo2 in liver cancer tissues, we examined the expression of Robo2 by western blot, and found that Robo2 in HCC tissues was markedly up-regulated compared with the paired adjacent liver tissues (Fig. 1C, D). Therefore, Robo2 in HCC tissues was elevated, which demonstrated that Robo2 may act as an oncogene in the tumorigenesis of HCC.

### Down-regulation of Robo2 constrains EMT and cell proliferation of HCC cells

**Figure 2:**
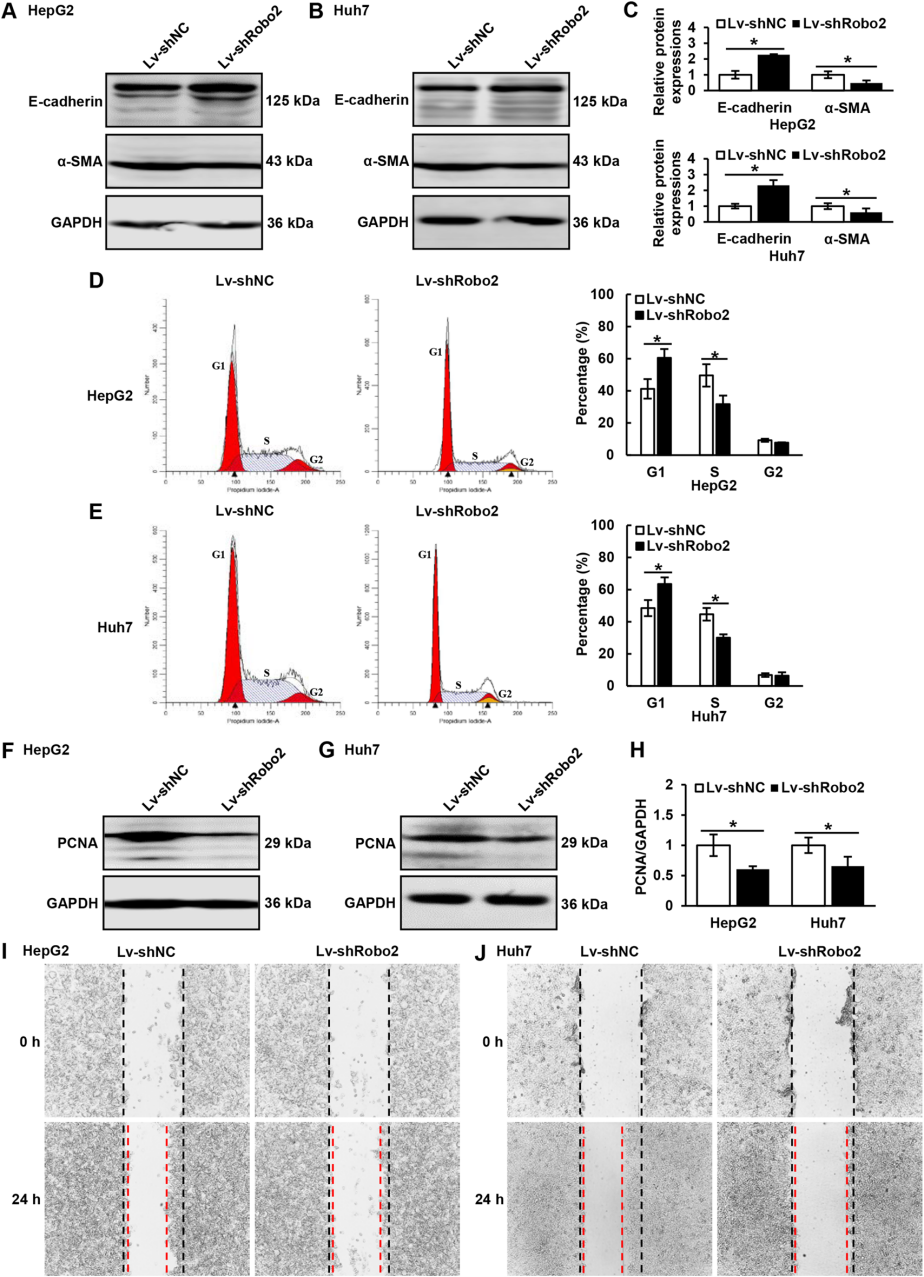
Effects of knocking down Robo2 on EMT and proliferation of hepatoma cells. (**A**–**C**) The expression of E-Cadherin and α-SMA in HepG2 and Huh7 cells was detected by Western blot. (**D**, **E**) The cell cycle changes after knocking down Robo2 were detected by flow cytometry. (**F**–**H**) The protein expression level of PCNA, a proliferation marker, was detected by Western blot. (**I**, **J**) The scratch healing experiment was carried out in Robo2-knock-down hepatoma cells. E-Cadherin, α-SMA and PCNA protein levels (normalized to GAPDH) were measured by scanning densitometry. *P < 0.05 *vs* indicated groups. The samples derive from the same experiment and that blots were processed in parallel. Original blots are presented in Supplementary Fig. 3.

EMT and cell proliferation play an important part in various malignant tumors. After successfully knocking down Robo2 (Supplementary Fig. 1A, B) in HepG2 and Huh7 cells, we found that the expression level of epithelial marker E-Cadherin was up-regulated while interstitial marker α-SMA was down-regulated (Fig. 2A–C). Next, we examined the changes of cell cycle after knocking down Robo2, and found that declined Robo2 led to the decrease of S-phase cells and the increase of G1-phase cells (Fig. 2D, E), which indicated that knocking down Robo2 could induce G1/S arrest of HCC cells. We also verified that down-regulated Robo2 suppressed the protein expression of PCNA (Fig. 2F–H) and the ability of wound healing (Fig. 2I, J). In a word, knock-down of Robo2 played an inhibitory role in EMT occurrence and cell proliferation of HCC cells.

### Knocking down Robo2 promotes the apoptosis in HCC cells

**Figure 3:**
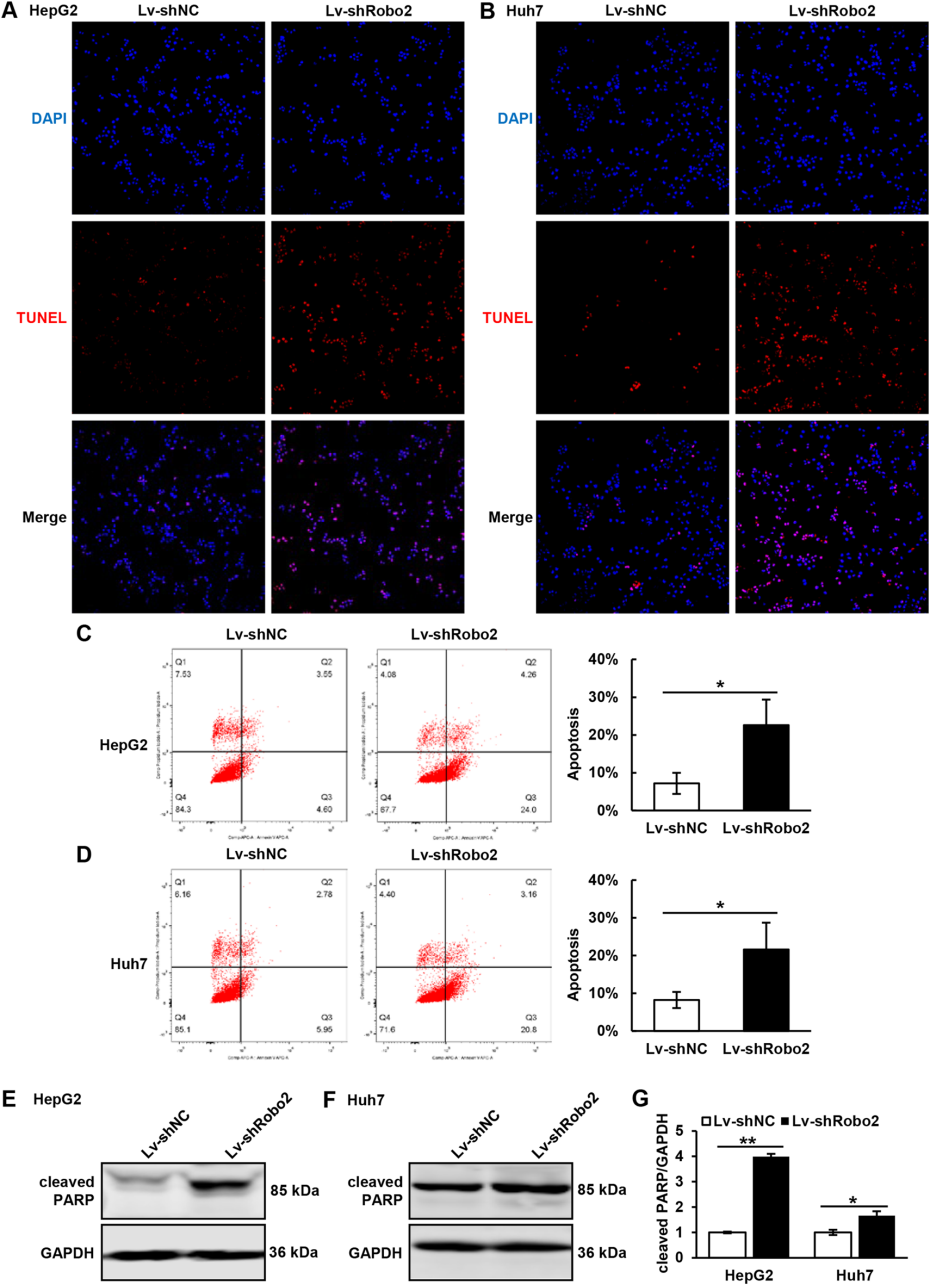
Function of Robo2 deficiency on the apoptosis of HCC cells. (**A**, **B**) TUNEL staining was performed in HepG2 and Huh7 cells. (**C**, **D**) Annexin V-APC and PI double staining were achieved in HCC cells by flow cytometry to detect apoptosis. (**E**–**G**) Western blot was used to detect the expression of cleaved-PARP, an apoptosis marker. Cleaved-PARP protein levels (normalized to GAPDH) were measured by scanning densitometry. *P < 0.05, **P < 0.01 *vs* indicated groups. The samples derive from the same experiment and that blots were processed in parallel. Original blots are presented in Supplementary Fig. 4.

We detected the apoptosis by TUNEL staining in HepG2 and Huh7 cells, and proved that knocking down Robo2 could induce obvious apoptosis (Fig. 3A, B). Cell apoptosis was further certified by flow cytometry, which was consistent with TUNEL staining, and the consequences demonstrated that inhibition of Robo2 could increase cell apoptosis (Fig. 3C, D). At last, the apoptosis marker protein cleaved PARP was reduced examined by western blot assay in HepG2 and Huh7 cells with Robo2 knocking down (Fig. 3E–G). All the above results confirmed that inhibition of Robo2 could enhance the apoptosis of hepatoma cells.

### YB-1 is a direct target interacting with Robo2

**Figure 4:**
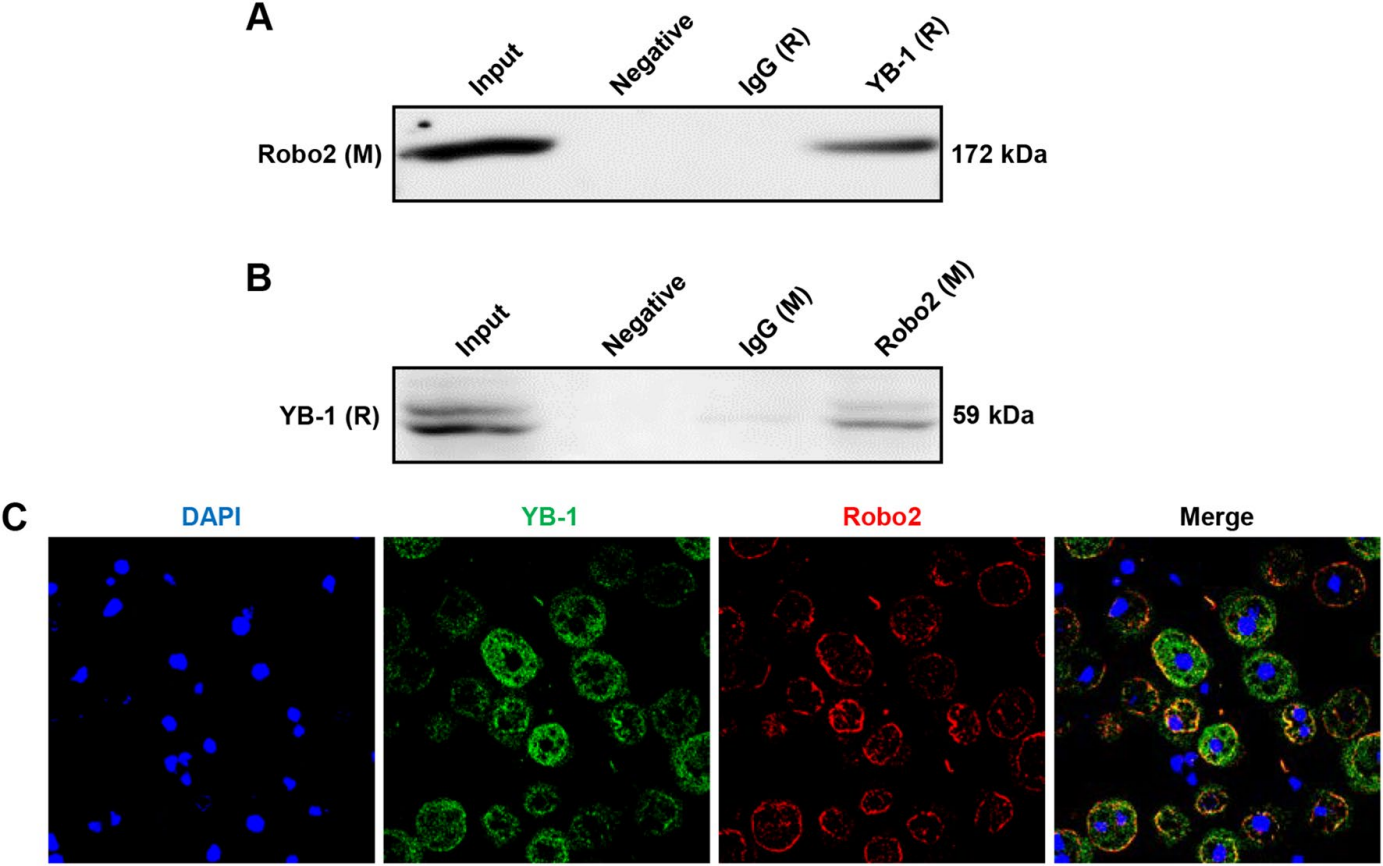
Robo2 interacts with YB-1. Co-IP was conducted by utilizing YB-1 antibody (rabbit derived) and followed by Western blot using Robo2 antibody (mouse derived) (**A**); simultaneously, Co-IP using Robo2 antibody and followed by Western blot using YB-1 antibody (**B**) was carried out. (**C**) Co-localization between Robo2 and YB-1 in HepG2 cells was detected by double immunofluorescence staining (100×).

To further explore the molecular mechanism of Robo2 in the progression of HCC, our research group previously had performed RNA sequencing in YB-1 knocked-down Huh7 cells, and revealed that the expression level of Robo2 was also down-regulated after YB-1 was knocked down, and it has been confirmed that YB-1 promoted the occurrence of HCC^14^. Therefore, we attempted to verify whether Robo2 interacted with YB-1. Firstly, we proved whether there was interaction between Robo2 and YB-1 by Co-IP assay, and found that Robo2 could be immunoprecipitated by rabbit YB-1, but no target protein could be immunoprecipitated by control rabbit IgG and negative control (Fig. 4A). Accordingly, YB-1 was immunoprecipitated by mouse Robo2, while the control mouse IgG and negative control did not precipitate YB-1(Fig. 4B), which confirmed that Robo2 could interact with YB-1. Then, we conducted immunofluorescence co-localization of Robo2 and YB-1, which demonstrated that Robo2 and YB-1 were co-located and mainly accumulated on cell membrane (Fig. 4C, yellow signal). All the above results confirmed that there was a direct interaction between Robo2 and YB-1.

### YB-1 offsets the suppression of cell proliferation and EMT induced by knocking down Robo2

**Figure 5:**
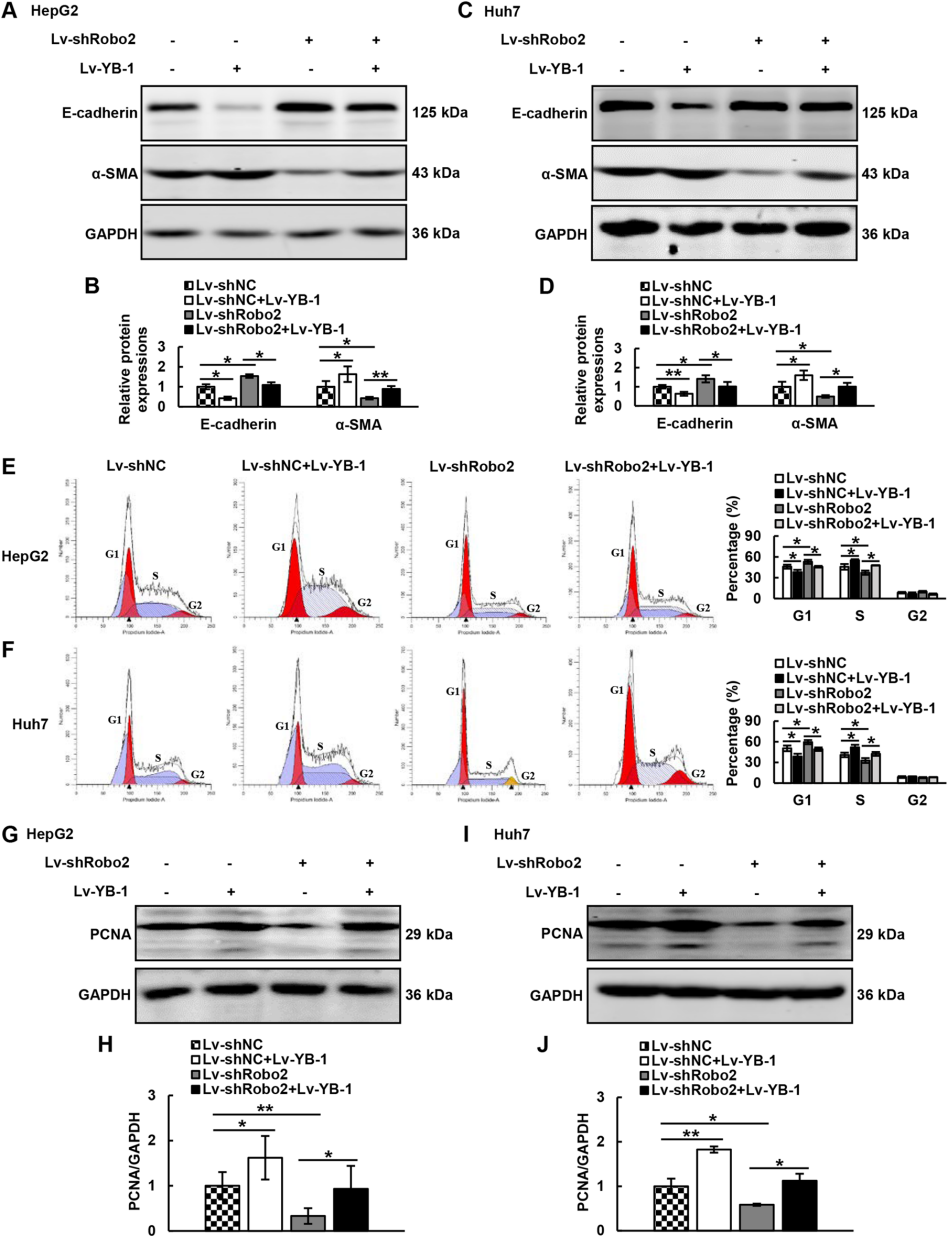
Robo2 regulates EMT generation and proliferation by YB-1. (**A**–**D**) The protein expression levels of E-Cadherin and α-SMA in Lv-shNC + Lv-NC, Lv-shNC + Lv-YB-1, Lv-shRobo2 + Lv-NC and Lv-shRobo2 + Lv-YB-1 groups of hepatoma cells were detected by Western blot. (**E**, **F**) Cell cycle changes in Lv-shNC + Lv-NC, Lv-shNC + Lv-YB-1, Lv-shRobo2 + Lv-NC and Lv-shRobo2 + Lv-YB-1 groups were examined by flow cytometry. (**G**–**J**) Western blot was exploited to detect the expression of PCNA in the above four groups of HepG2 and Huh7 cells. E-Cadherin, α-SMA and PCNA protein levels (normalized to GAPDH) were measured by scanning densitometry. *P < 0.05, **P < 0.01 *vs* indicated groups. The samples derive from the same experiment and that blots were processed in parallel. Original blots are presented in Supplementary Fig. 5.

To further elucidate the interaction mechanism between Robo2 and YB-1, we conducted rescue studies in HepG2 and Huh7 cells which knocked down Robo2 and/or over-expressed YB-1 (Supplementary Fig. 1C, D). Knocking down Robo2 restrained the occurrence of EMT, while over-expression of YB-1 had the inverse effect, and over-expression of YB-1 could weaken the inhibitory effect of knocking down Robo2 on EMT (Fig. 5A–D). Next, we detected the cell cycle in HCC cells with Robo2 knocking down and/or YB-1 over-expressing by flow cytometry, and verified that knocking down Robo2 caused G1 phase cells to increase and S phase cells to decrease; over-expression of YB-1 alone could increase the percentage of S-phase cells and decrease the percentage of G1-phase cells; and over-expression of YB-1 further weakened the cell cycle change induced by knocking down Robo2 (Fig. 5E, F), which indicated that YB-1 suppressed G1/S cell cycle arrest induced by knocking down Robo2 in HCC cells. To further prove the effect of YB-1 on the inhibition of cell proliferation produced by down-regulated Robo2, the results showed that knocking down Robo2 could prohibit the expression of PCNA in HepG2 and Huh7 cells, while up-regulated YB-1 reduced the inhibition function of Robo2 on PCNA expression (Fig. 5G–J). These consequences all confirmed that YB-1 could weaken the inhibitory effect of knocking down Robo2 on EMT and cell proliferation.

### Overexpression of YB-1 can reverse the apoptosis of hepatoma cells triggered by down-regulation of Robo2

**Figure 6:**
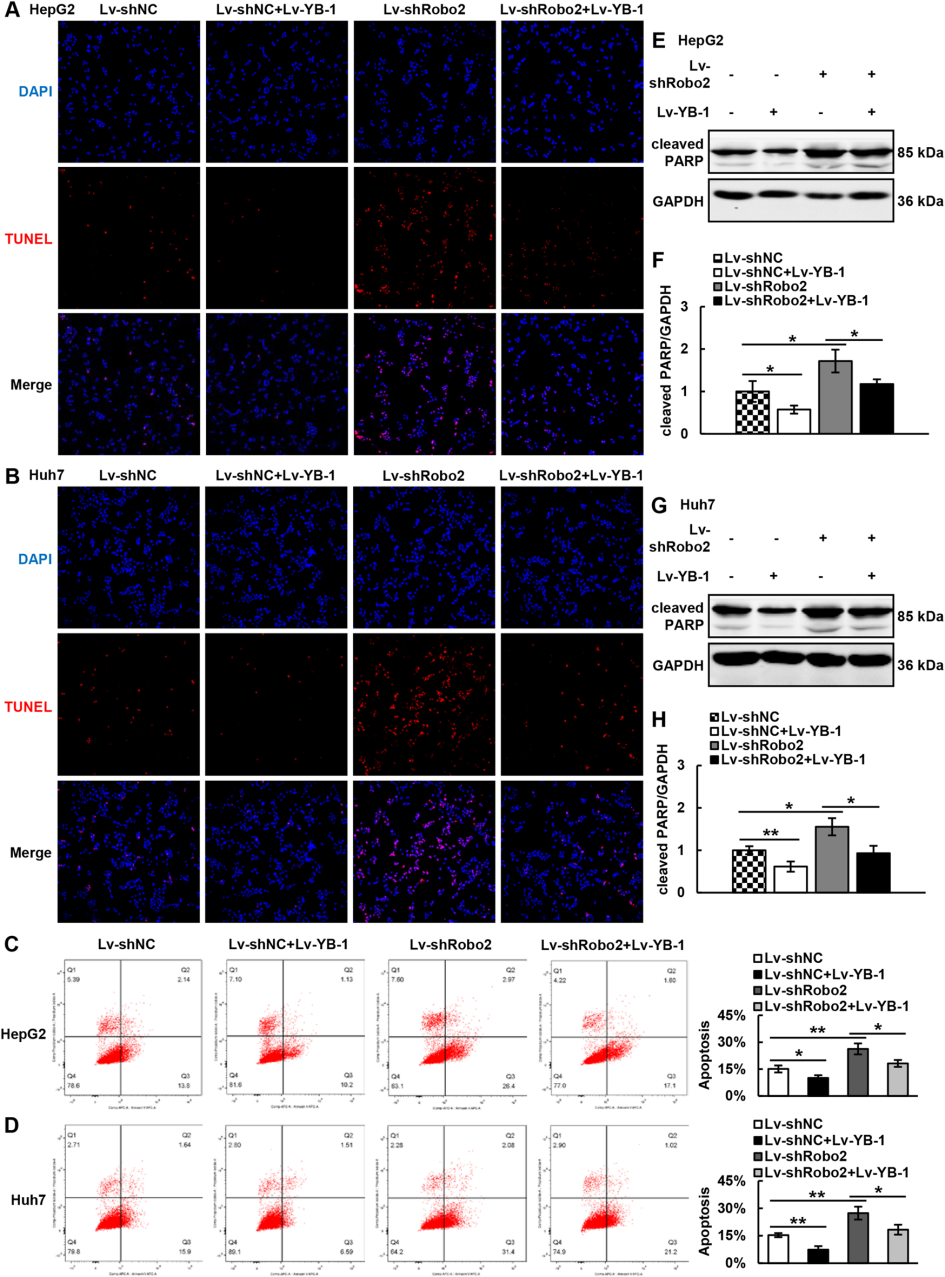
Robo2 regulates cell apoptosis by interacting with YB-1. (**A**, **B**) Apoptosis was tested by TUNEL staining in Lv-shNC + Lv-NC, Lv-shNC + Lv-YB-1, Lv-shRobo2 + Lv-NC and Lv-shRobo2 + Lv-YB-1 groups of HepG2 and Huh7 cells. (**C**, **D**) Annexin V-APC and PI double staining was utilized to detect the apoptosis. (**E**–**H**) Western blot was applied to test the expression of cleaved-PARP in the above four groups of HCC cells. Cleaved-PARP protein levels (normalized to GAPDH) were measured by scanning densitometry. *P < 0.05, **P < 0.01 *vs* indicated groups. The samples derive from the same experiment and that blots were processed in parallel. Original blots are presented in Supplementary Fig. 6.

Meanwhile, we detected the apoptosis in the hepatoma cells, TUNEL staining showed that knock-down Robo2 could increase the apoptosis, while over-expression of YB-1 could counteract the upsurge of apoptosis caused by down-regulation of Robo2 (Fig. 6A, B). Flow cytometry revealed consistent results with TUNEL staining (Fig. 6C, D). At last, we further detected the apoptosis marker protein cleaved PARP, and identified that knocking down Robo2 raised cleaved PARP, but when combined with YB-1 overexpression, the level of cleaved PARP decreased to normal (Fig. 6E–H). The above findings all confirmed that YB-1 restrained the hepatoma cell apoptosis induced by the deficient expression of Robo2.

## Discussion

So far, researchers have mainly studied the role of Robo2 in pancreatic cancer and liver fibrosis. To our knowledge, we first explored the role and mechanism of Robo2 in HCC. In this study, we found that the expression level of Robo2 in human HCC tissues was significantly higher than that in normal liver tissues adjacent to cancer, and down-regulation of Robo2 could suppress the EMT and proliferation of hepatoma cells and stimulate the apoptosis of HepG2 and Huh7 cells. More importantly, Robo2 facilitated the onset of HCC by modulating YB-1.

Robo protein is a single transmembrane receptor of immunoglobulin superfamily. At present, four types of Robo proteins (Robo1, Robo2, Robo3 and Robo4) have been discovered in mammals. The basic structure of Robo protein includes five immunoglobulin-like domains, three fibronectin type III domains, one transmembrane domain and an intracellular domain^7,8^. The extracellular ligand binding domain of Robo protein is highly conserved from Drosophila melanogaster to human, and the intracellular domain has no obvious catalytic activity and little homology, but there are four short conserved motifs (CC0, CC1, CC2 and CC3) in the cell, which are deemed to binding sites for Robo protein to interact with diverse intracellular proteins or protein kinases^7,8^.

Previous studies have proved that Robo protein played an important role in pancreatic cancer, glioblastoma and other malignant tumors, for example, compared with the healthy control group, Slit/Robo signaling pathway in low-grade and early-stage endometrial cancer was out of balance at the level of protein group and transcriptome^15^; silymarin prohibited the malignant biological behavior of prostate cancer cells by activating Slit2/Robo1 pathway and inhibiting the expression of C-X-C chemokine receptor type 4 (CXCR4)^16^; compared with normal and non-cancerous intraductal proliferative lesions, Robo3 in invasive ductal carcinoma of breast decreased^17^; down-regulation of Robo4 could stimulate the malignant phenotypic changes of pancreatic cancer cells by facilitating matrix metalloproteinase-9 (MMP-9), and it was relevant to the poor prognosis of pancreatic cancer^18^. There were still few studies on the role of Robo protein in chronic liver diseases such as liver fibrosis: Yuen et al.^19^ found that Robo1 was involved in the pathogenesis and progression of renal fibrosis in mice with ischemia and obstructive renal injury; in addition, Chang et al.^20^ verified that Robo1 expedited liver injury and liver fibrosis by stimulating HSCs in CCl4-induced mouse liver fibrosis model, and they further proved that inhibiting Slit2-Robo1 signaling pathway suppressed the activation of HSCs by blocking Smad2/3 and PI3K/Akt signaling pathways. Our research group previously also substantiated that sTREM-1/Robo2 strengthened HSCs activation and the occurrence of hepatic fibrosis through PI3K/Akt and Smad2/3 signaling pathways^13^.

However, there were few researches about Robo protein in HCC at present, such as silymarin prohibited apoptosis and metastasis of HepG2 cells by down-regulating Slit2/Robo1 signaling pathway^21^; Robo1 was over-expressed in cancer tissues of HCC patients, and maybe a potential therapeutic target for HCC^22^; Slit-Robo1 signaling pathway accelerated angiogenesis of malignant melanoma and neutralizing antibody of Robo1 significantly inhibited tumor growth^23^. Robo2 belongs to the same family of Robo proteins, so we speculate that Robo2 should have a similar function to other protein members. Related studies on Robo2 in HCC verified that the expression level of Robo2 could predict the alpha fetoprotein (AFP)-dependent subgroup of liver cancer cell lines, and Robo2 was considerably up-regulated in hepatoma cell lines with high AFP background^24^. At the same time, previous studies had confirmed that canceration was a necessary step for malignant transformation: insensitivity to growth inhibition signals, escape of cell apoptosis, unlimited replication potential, self-sufficiency of growth signals, continuous angiogenesis, tissue infiltration and metastasis, redistribution of energy metabolism, escape of cellular immunity and so on^25^. These consequences were coincident with our study, that is, the expression level of Robo2 in HCC tissues was significantly elevated, and it played a role in promoting liver cancer.

YB-1 is a multifunctional protein consisting of 324 amino acids, and it is one of the members of Y-box binding protein family^26^. Previous studies found that YB-1 in pancreatic cancer, colorectal cancer, breast cancer, melanoma and sarcoma was dramatically raised^27–29^; studies had also shown that the expression of YB-1 in HCC was closely related to the acquisition of stem cell characteristics, drug resistance, migration and proliferation, etc.^27^. In addition, our team’s previous research found that YB-1 augmented the sorafenib resistance in HCC cells through PI3K/Akt signaling pathway^14^. More importantly, we carried out transcriptome sequencing in the YB-1 knocked-down Huh7 cells formerly, and found that Robo2 was synonymously declined after YB-1 was knocked down^14^. This study further demonstrated that YB-1 interacted with Robo2 through Co-IP, immunofluorescence co-staining and rescue study, and that Robo2 played a part in the pathogenesis of HCC by regulating YB-1. However, since Robo2 had a long coding DNA sequence (4185 bp), its overexpression was difficile to realize. Simultaneously, foregoing studies only detected the conversions of downstream signaling pathways and related proteins after knocking out Robo2^13,20^, so we studied the performance of Robo2 in HCC by knocking out its expression.

In summary, we innovatively explore the role and mechanism of Robo2 in HCC. This research certifies that down-regulation of Robo2 can facilitate the EMT and proliferation, and constrain the apoptosis of HCC cells by regulating YB-1, which provided a basis for the pathogenesis of HCC and a potential new target for the clinical therapy of HCC.

## Materials and methods

### Patients and HCC samples

The HCC and matched adjacent normal tissues of 6 HCC patients who underwent surgical resection from 09/2020 to 11/2020 were collected. All the HCC patients included in the study met the clinical and histological diagnostic criteria for HCC in China^30^, and invalids with carcinoma recurrence or other organ malignancies were ruled out. Histological evaluation and diagnosis were conducted by two seasoned pathologists. After the tissues were obtained, they were fixed in 4% paraformaldehyde (Beyotime Biotechnology Co., Shanghai, China) or instantly stored at − 80 °C. All patients who participated in the study signed the informed consent forms. The study had been authorized by the Ethics Committee of the First Hospital of Hebei Medical University (Approval Letter No.: 20220805), and the study protocol conforms to the ethical guidelines of the 1975 Declaration of Helsinki (6th revision, 2008).

### Cell culture

Human HCC cell lines (HepG2 and Huh7) were acquired from the American Type Culture Collection (ATCC, Manassas, VA, USA). Cells were cultivated in Dulbecco’s modified Eagle’s medium (DMEM; Gibco BRL, Grand Island, NY, USA) containing 10% fetal bovine serum (FBS, HyClone, South Logan, UT, USA). Robo2 knock-down was accomplished by using lentiviral vectors harboring Robo2 specific shRNA (Lv-shRobo2, Shanghai Genechem Co., Ltd, Shanghai, China), and we tested the knock-down efficiency by Western blot (Supplementary Fig. 1A, B). Over-expression of YB-1 was achieved by transfection of lentivirus-YB-1 (Lv-YB-1; Shanghai GenePharma Co., Ltd., Shanghai, China), the overexpression efficiency was displayed in Supplementary Fig. 1C, D.

### Western blot analysis

Primary antibodies used in our study were Robo2 (diluted 1:50; Santa Cruz Biotechnology, Santa Cruz, CA, USA), YB-1 (diluted 1:200; Shanghai Abways Biotechnology Co., Ltd., Shanghai, China), α-smooth muscle actin (α-SMA; diluted 1:200; Abways), E-Cadherin (diluted 1:1000; Proteintech Group Inc., Wuhan, China), proliferating cell nuclear antigen (PCNA; diluted 1:1000; Abways), PARP (diluted 1:1000; Cell Signaling Technology, Danvers, MA, USA) and glyceraldehyde-3-phosphate dehydrogenase (GAPDH; diluted 1:1000; Abways). We lysed the cells or tissues by utilizing radioimmunoprecipitation assay buffer (Beyotime Biotechnology Co.) for total protein extraction. The proteins were quantified, denatured and electrophorized in SDS–polyacrylamide gel electrophoresis. After transferred, the polyvinylidene difluoride (PVDF) membranes were sealed with 5% milk and hatched with the primary antibody at 4 ℃ overnight. The next day, the membranes were incubated with fluorescent coupled secondary antibodies (LI-COR Biosciences, Lincoln, NE, USA). Finally, the PVDF membranes were scanned by the Odyssey infrared imaging system, and the protein bands were quantified by ImageJ software. Objective protein bands were standardized against glyceraldehyde-3-phosphate dehydrogenase (GAPDH). Regarding the absence of original blots of adequate length, we provide the following explanations: (1) we cropped the blots before antibody incubation, resulting in the target protein and reference protein not being displayed on the same blot, we assure that the samples derive from the same experiment, and the blots were processed in parallel; (2) this study was conducted concurrently with other western blot experiments in our research team, as a result, some of the cropped bands are actually related to other experiments, making it inconvenient for us to display them; (3) our study employed the Odyssey infrared imaging system for blot scanning, the scanning settings led to the blots' edges being smaller than the original bands, causing partial bands' edge regions not to be fully displayed.

### Histological and immunohistochemical (IHC) analysis

Tissues were fixed with 4% paraformaldehyde, embedded in paraffin and sectioned. Then, the sections were stained with hematoxylin and eosin (HE) and IHC according to the previously described standard scheme^31^. We utilized Robo2 antibody (diluted 1:200; Cell Signaling Technology) for IHC staining, and IHC images were collected by the upright microscope.

### Cell cycle analysis

We collected and fixed the cells in 70% ethanol, and then added PI/RNase Staining Solution (Beyotime Biotechnology Co.) and incubated them at 37 °C in the dark for 30 min. Samples were analyzed by a FACSVerse flow cytometer, and data were processed by ModFit 5.0 software.

### Wound healing assay

HepG2 and Huh7 cells with Robo2 knocking down were inoculated into the 6-well plates and the confluent cells were scraped with a 200 μL sterile pipette tip when the cell density reached 70%. Next, the cells were cultured in serum-free DMEM medium and photographed at 0 h and 24 h after scratching.

### Apoptosis assay

We used Annexin V Apoptosis Detection Kit (Invitrogen, Carlsbad, CA, USA) to examine the cell apoptosis. In brief, the collected cells were added to the binding buffer containing 2.5 μL of Annexin V-APC and 5 μL of propidium iodide (PI) and incubated in the dark for 20 min at room temperature. Samples were analyzed by flow cytometry. In addition, we employed a one-step terminal deoxynucleotidyl transferase dUTP nick end labeling (TUNEL) apoptosis detection kit (Beyotime Biotechnology Co.) to detect apoptosis. In this study, representative images were taken by a laser scanning confocal microscope, and TUNEL-positive cells were counted and recorded.

### Co-immunoprecipitation (Co-IP)

The total cell protein was extracted by utilizing the cold lysis buffer. The protein samples were incubated with protein A/G agarose beads (Beyotime Biotechnology Co.) to preclear nonspecific binding. Then, the precleared lysate and primary antibodies were incubated overnight at 4 ℃ with rotation gently, and protein A/G magnetic beads were added and incubated for 2 h the next day. The primary antibodies applied in our research included mouse anti-Robo2 (diluted 1:20; Santa Cruz), rabbit anti-YB-1 (1:100; Abways), normal rabbit IgG (1:500; Beyotime Biotechnology Co.) and normal mouse IgG (1:500; Beyotime Biotechnology Co.). After incubation, we re-enriched the beads with a magnetic stand and washed with IP washing buffer. The beads were then resuspended in 2 × SDS loading buffer (Beyotime Biotechnology Co.) and boiled for 10 min to release the bound proteins, which were subsequently electrophoretic separated by SDS-PAGE, followed by western blot analyses as described above.

### Immunofluorescence (IF)

Cells grown on coverslips were fixed in 4% paraformaldehyde, permeabilized in 0.5% TritonX-100 (Beyotime Biotechnology Co.) and then we blocked the cells in 5% bovine serum albumin (Solarbio Science & Technology Co., Ltd., Beijing, China). Subsequently, we incubated the cells with mouse anti-Robo2 (diluted 1:50; Santa Cruz) and rabbit anti-YB-1 (diluted 1:200; Abways) overnight at 4 ℃. At the next day, the secondary antibodies (Cy3 anti-mouse IgG, FITC anti-rabbit IgG, Beyotime Biotechnology Co.) were applied. Finally, we stained the nucleus with 4′6′-diamidino-2-phenylindole dihydrochloride (Solarbio), and took representative pictures with the laser scanning confocal microscope.

### Statistical analysis

The data of normal distribution were expressed as mean and standard deviation, while the data of non-normal distribution were expressed as median and interquartile spacing. The differences between two groups were statistically analyzed by Student’s *t*-test. The differences among various groups were analyzed by one-way ANOVA. All statistical analyses were done by GraphPad Prism 8.3.0 and SPSS software version 19.0. All the experiments were repeated three times. *P*-values less than 0.05 were considered to be statistically significant.

### Ethics declarations

The study was reviewed and approved by the Ethics Committee of the First Hospital of Hebei Medical University (approval letter No.: 20220805).

### Supplementary Information

Supplementary Figures.

## Data Availability

The data supporting the findings of this study are available from the corresponding author upon reasonable request.
