# Supplementary material for: Downregulation of Roundabout guidance receptor 2 suppresses hepatocellular carcinoma progression by interacting with Y-box binding protein 1
Source: Sci Rep. 2024 Jan 31;14:2588. doi: 10.1038/s41598-024-53013-3 (PMC10830551; doi:10.1038/s41598-024-53013-3)
Supplement: Supplementary file 1 — Supplementary Figures. [file 41598_2024_53013_MOESM1_ESM.pdf]

Supplementary Fig. 1

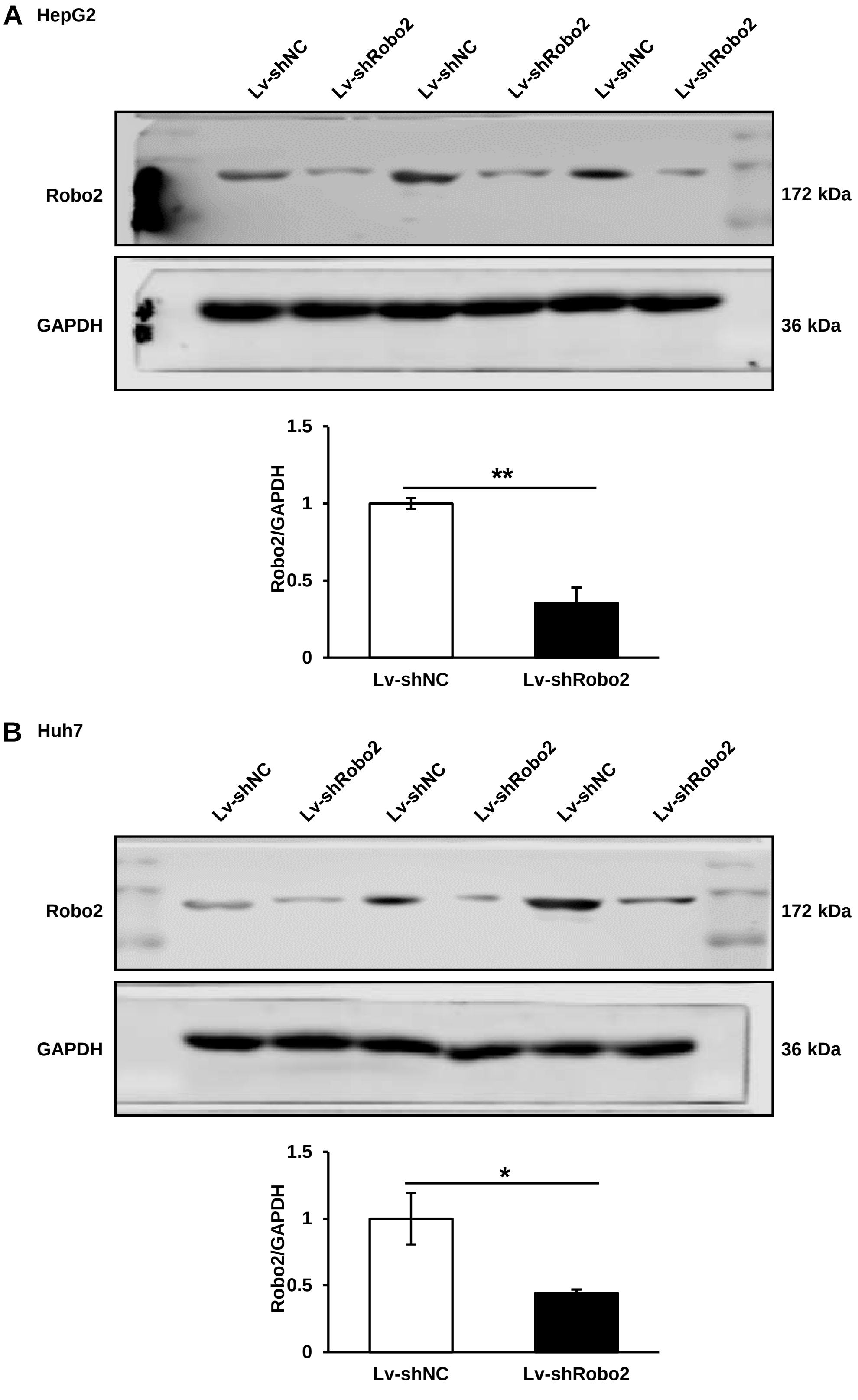

Supplementary Fig. 1

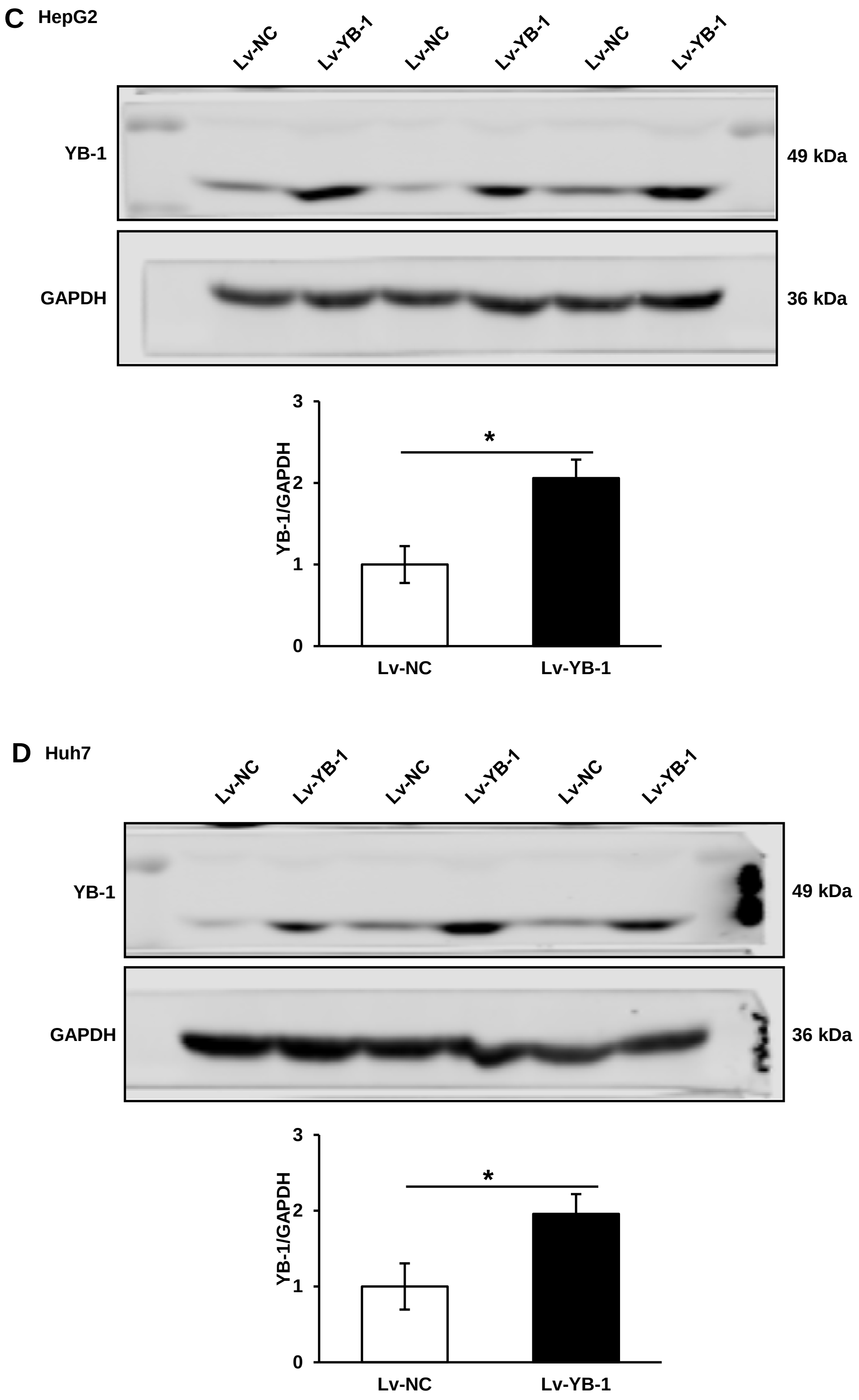

Supplementary Figure 1 Detection of the transfection efficiency of knock-down Robo2 and over-expression YB-1. (A-D) The transfection efficiency of knock-down Robo2 and over-expression YB-1 in HepG2 and Huh7 cells was detected by Western blot assay. Robo2 and YB-1 protein levels (normalized to GAPDH) were measured by scanning densitometry. * P<0.05, ** P<0.01 *vs* indicated groups. The samples derive from the same experiment and that blots were processed in parallel.

Supplementary Fig. 2

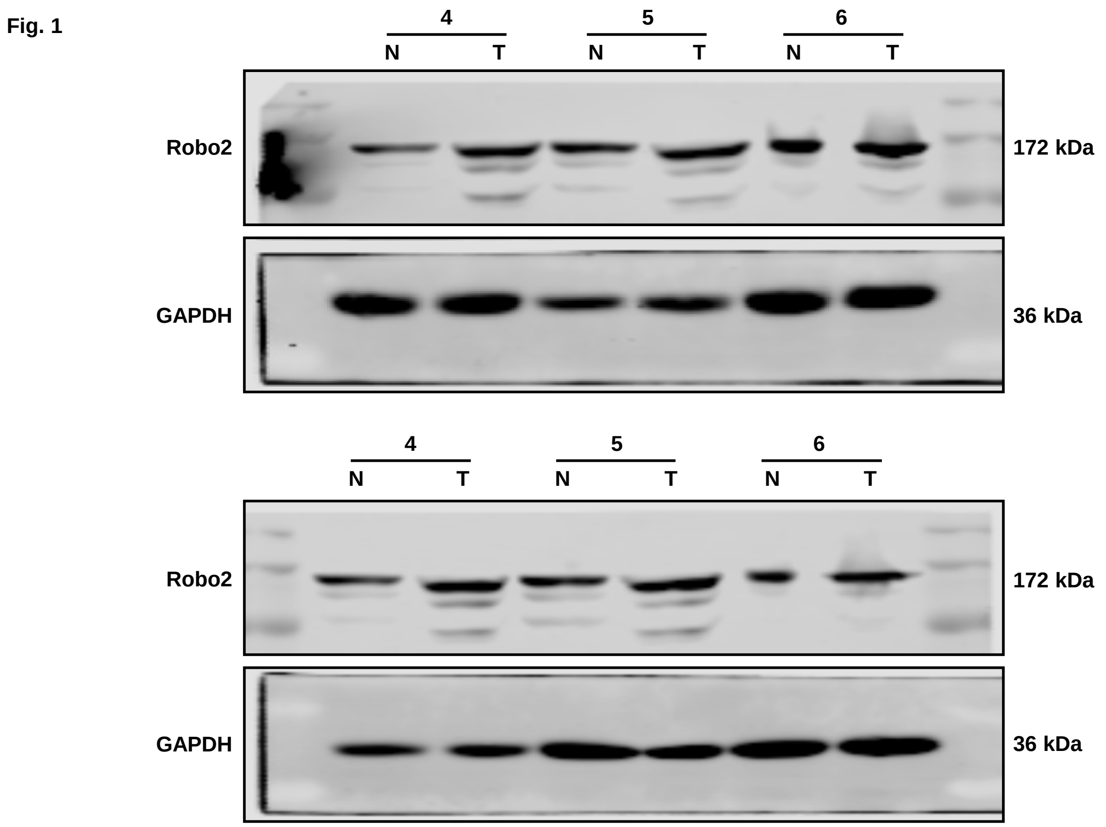

Supplementary Figure 2 Original blots in Fig. 1. The samples derive from the same experiment and that blots were processed in parallel.

Supplementary Fig. 3

Fig. 2

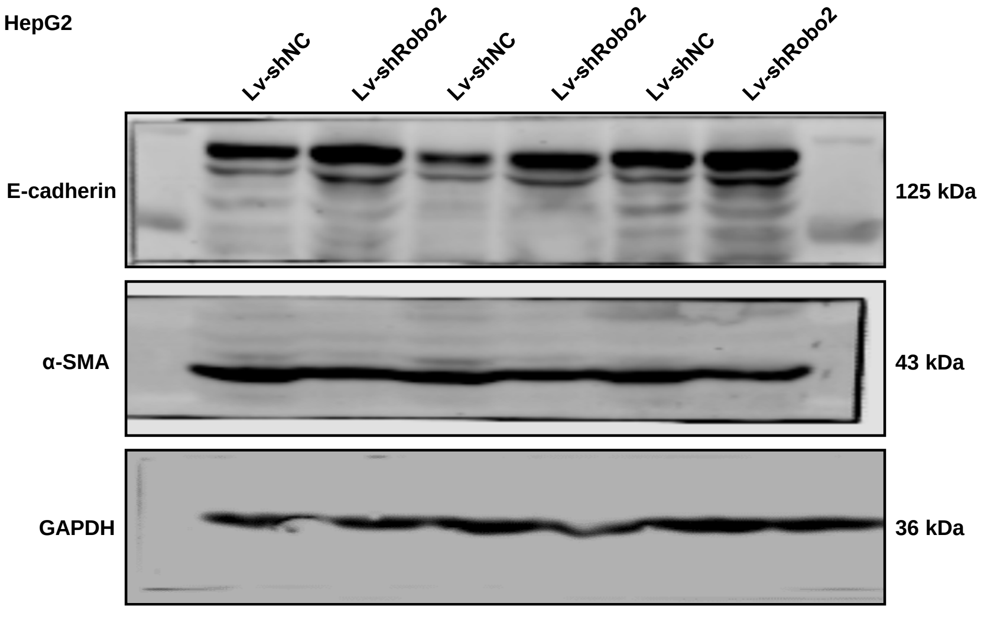

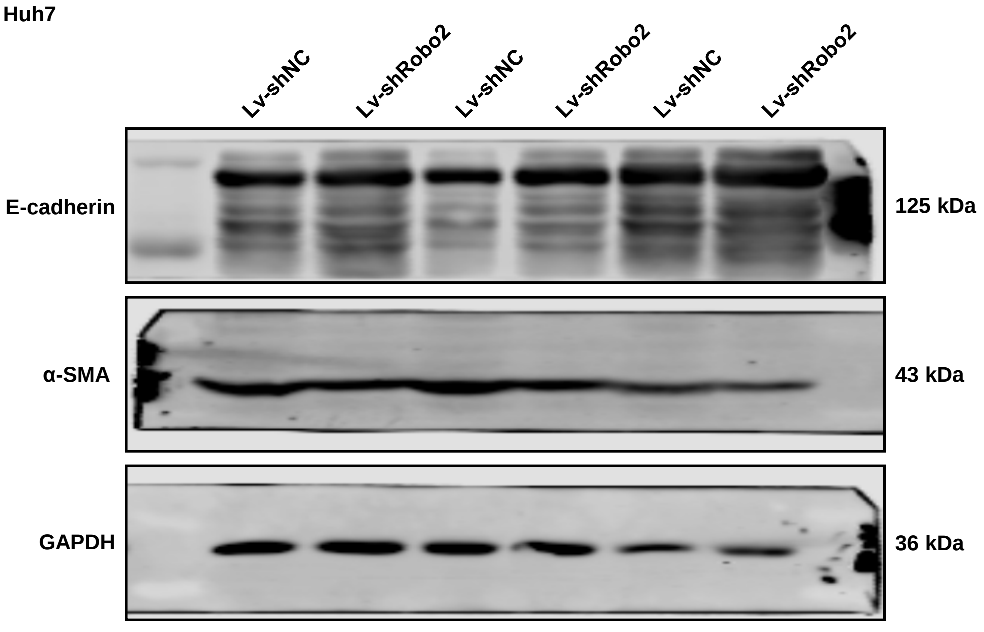

Supplementary Fig. 3

Fig. 2

HepG2

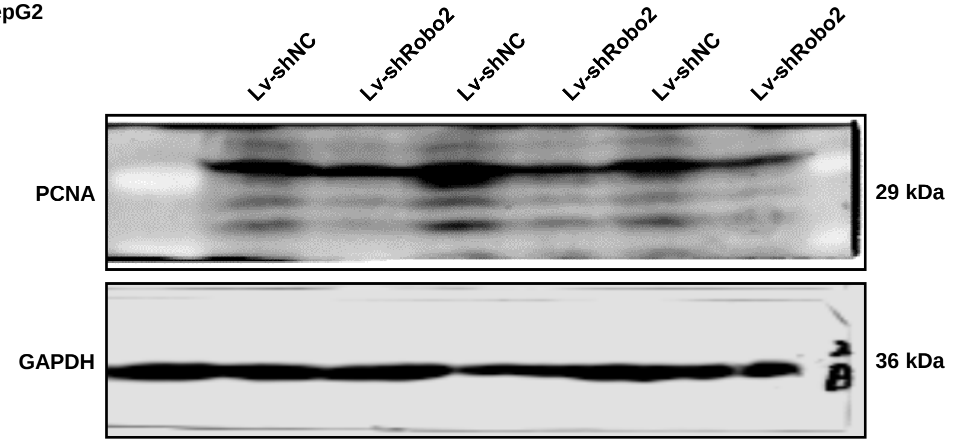

Huh7

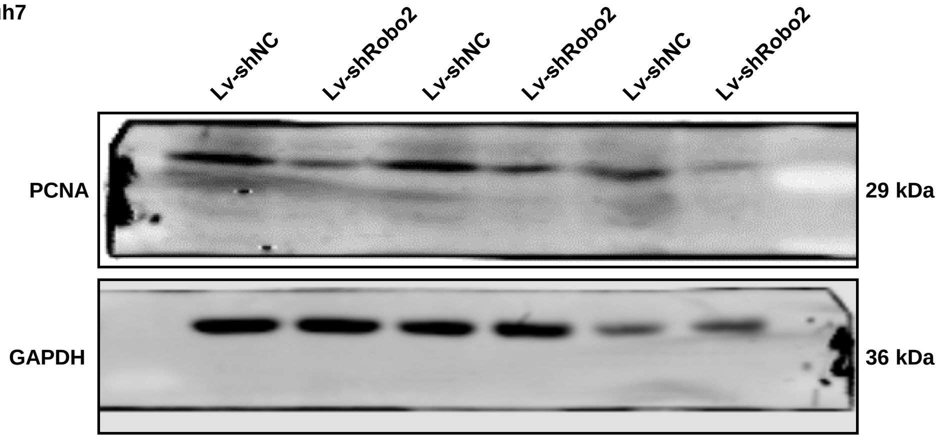

Supplementary Figure 3 Original blots in Fig. 2. The samples derive from the same experiment and that blots were processed in parallel.

Supplementary Fig. 4

Fig. 3

HepG2

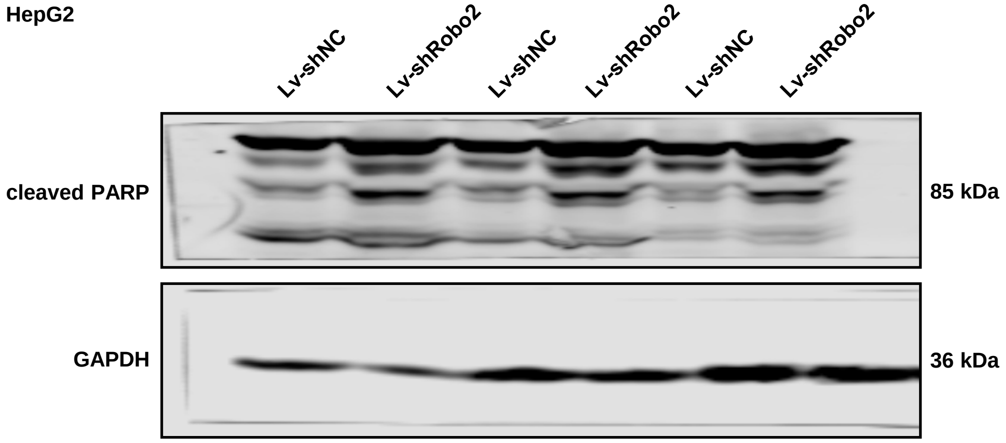

Huh7

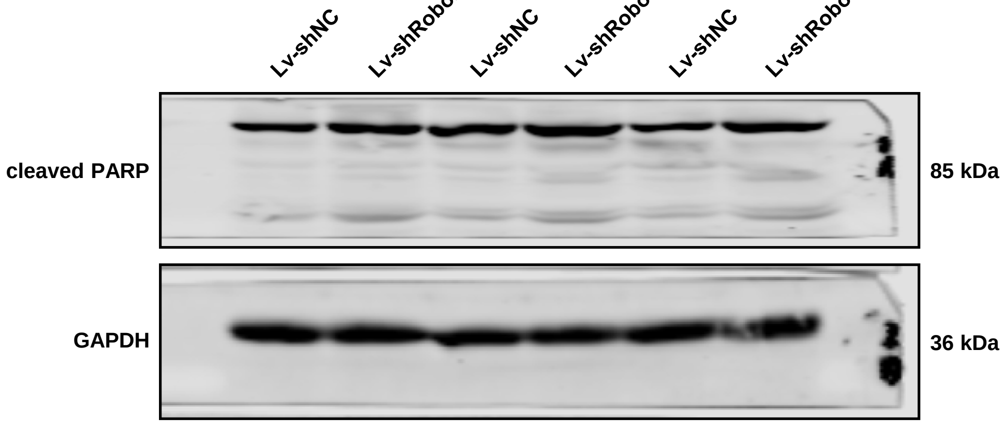

Supplementary Figure 4 Original blots in Fig. 3. The samples derive from the same experiment and that blots were processed in parallel.

Supplementary Fig. 5

Fig. 5

HepG2

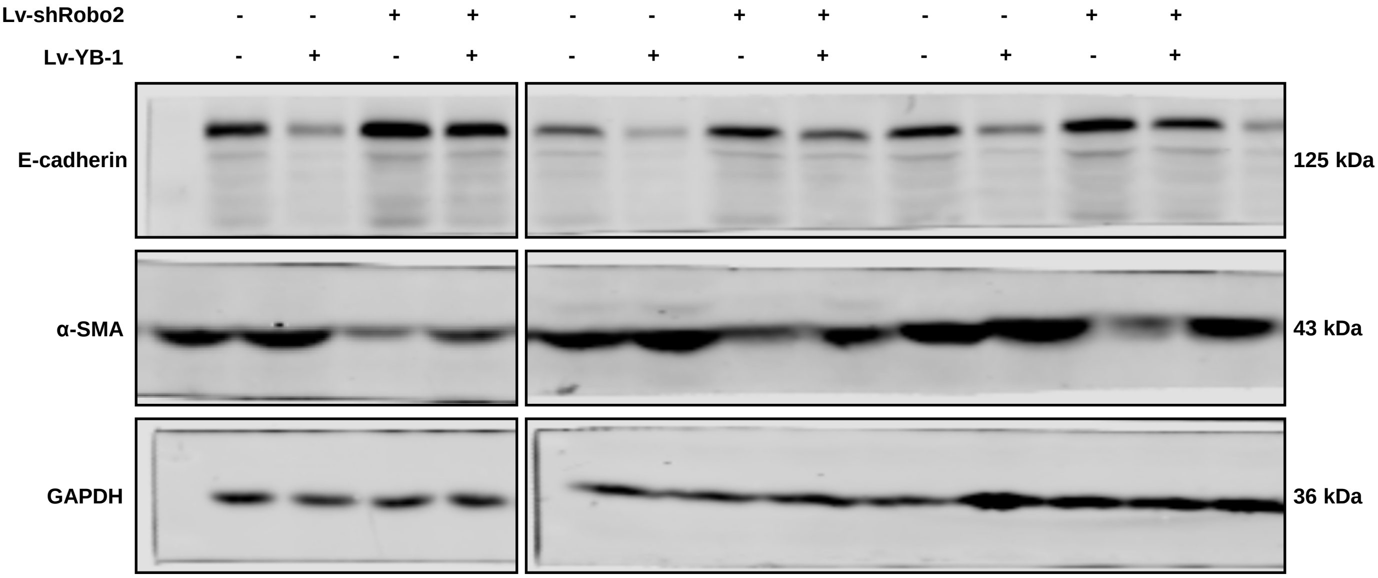

Huh7

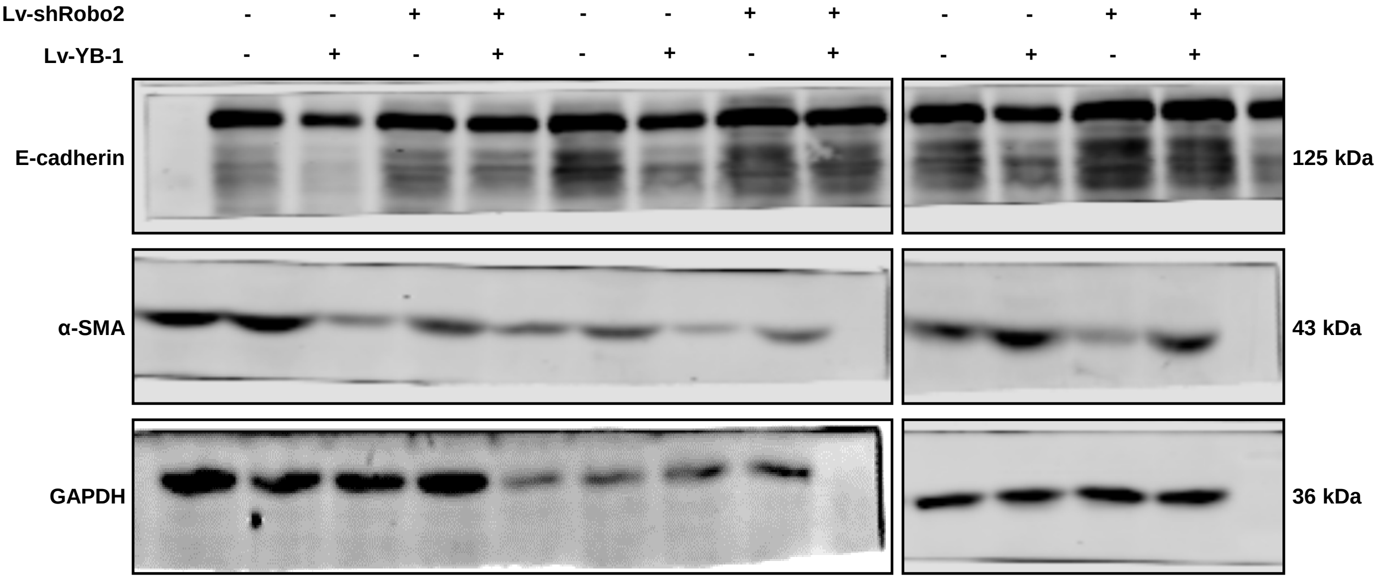

Supplementary Fig. 5

Fig. 5

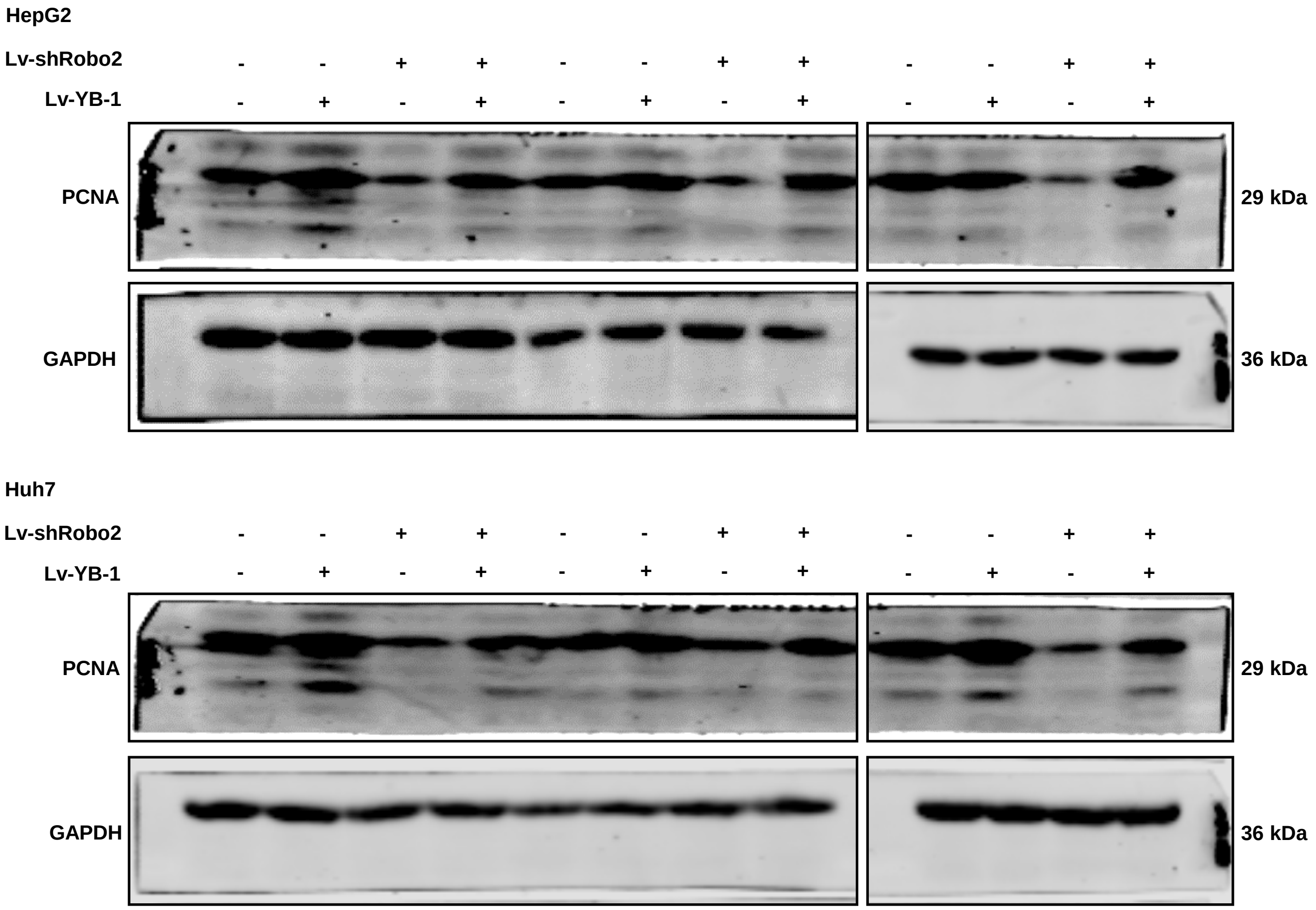

Supplementary Figure 5 Original blots in Fig. 5. The samples derive from the same experiment and that blots were processed in parallel.

Supplementary Fig. 6

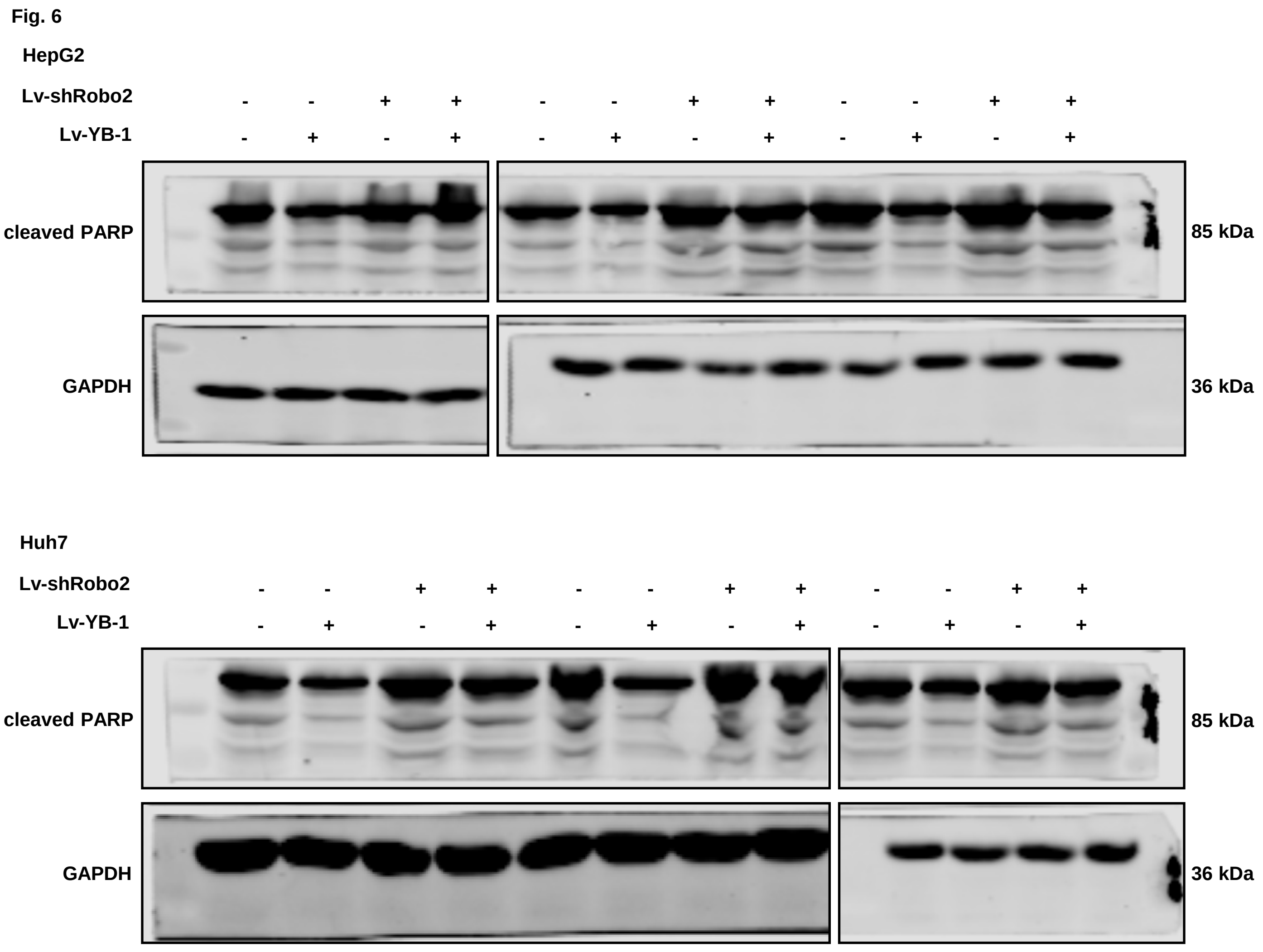

Supplementary Figure 6 Original blots in Fig. 6. The samples derive from the same experiment and that blots were processed in parallel.
